# Inhibition of the right dlPFC by theta burst stimulation does not alter sustainable decision-making

**DOI:** 10.1038/s41598-019-50322-w

**Published:** 2019-09-25

**Authors:** Benedikt P. Langenbach, Thomas Baumgartner, Dario Cazzoli, René M. Müri, Daria Knoch

**Affiliations:** 1 University of Bern, Institute of Psychology, Department of Social Psychology and Social Neuroscience, Fabrikstrasse 8, 3012 Bern, Switzerland; 2 University Hospital Bern, Department of Neurology, University Neurorehabilitation, Freiburgstrasse 10, 3010 Bern, Switzerland; 3 Bern University Hospital and University of Bern, Department of Neurology, University Neurorehabilitation, Freiburgstrasse 10, Bern, Switzerland; 4 University of Bern, ARTORG Center for Biomedical Engineering Research, Gerontechnology and Rehabilitation Group, Murtenstrasse 50, Bern, Switzerland

**Keywords:** Cognitive control, Social behaviour, Social neuroscience, Human behaviour

## Abstract

Intergenerational sustainability is probably humankind’s most pressing challenge, exacerbated by the fact that the present generation has to incur costs in order to benefit future generations. However, people often fail to restrict their consumption, despite reporting strong pro-environmental attitudes. Recent theorising sees self-control processes as key component of sustainable decision-making and correlational studies support this view, yet causal evidence is lacking. Using TMS, we here disrupted an area known to be involved in self-control processes, the right dorsolateral prefrontal cortex (dlPFC), to provide causal evidence as to whether diminished self-control leads to less intergenerational sustainability. Participants then engaged in a behavioural economic paradigm to measure sustainable decision-making towards the next generation. This adequately powered study could not find an effect of inhibiting the right dlPFC on intergenerational sustainability. This result holds when controlling for a number of relevant covariates like gender, trait self-control, pro-environmental attitudes, or cortical thickness at the stimulation site. We seek to explain this result methodologically and theoretically, and speculate about other brain areas that could be more strongly related to intergenerational sustainability, e.g. the mentalising network.

## Introduction

Intergenerational sustainability is vital for the existence of humankind. Since global climate change poses a serious threat to the well-being of billions of people^1^, policy makers and environmental organisations are left with the challenging task of steering individual human behaviour towards more sustainable decisions^2^. However, ensuring the benefit of future generations would require humans to make sacrifices today. A 2015 Nature Climate Change review stresses the urgency to understand individuals’ cognitive processes that cause sustainable environmental decision-making in order to encourage it^3^. To this date, a large number of behavioural intervention campaigns focus on raising the level of awareness about climate problems. However, information campaigns promoting pro-environmental behaviour have been less effective than expected^4^, and most people already acknowledge the urgency to take action^5^. Given the gap between most people’s attitudes and the sustainability of their behaviour^6,7^, further raising the level of awareness about climate change might not be completely sufficient to encourage sustainable decision-making. Some environmental scientists addressed this issue by proposing a stronger focus on the “cognitive barriers”^8^ that hinder sustainable environmental decision-making – with *self-regulation capacity* being one potentially promising candidate of such a barrier^9–11^, although empirical evidence for its involvement in sustainable decision-making is rare. Self-regulation (or self-control; used interchangeably throughout this paper), refers to the human capacity to align one’s behaviour with a certain goal. In everyday life, this often involves the effortful inhibition of alternative actions that are more tempting^12^. For example, if one chooses to use less warm water, self-control efforts will likely be required to resist the temptation of enjoying a long, relaxing shower. Like here, sustainable decision-making presumably requires a substantial amount of self-control in many situations^11^: Consider the example of a person who is concerned about the effects global climate change will have on future generations and who wants to cut down her CO_2_ emissions. To take appropriate measures (e.g., eating less meat; using public transport instead of the car; passing on a weekend trip by plane), said person must sacrifice her own comfort for the sake of a future generation – a process that might not be achieved without sufficient self-regulation capacities. Arguably, self-control is especially important when a pro-environmental action requires the present generation to sacrifice their own comfort for a future generation. Importantly, this intergenerational sustainability dilemma differs substantially from other more extensively studied intertemporal choice situations^13^: When people can choose a larger future reward over a smaller immediate one, the decision-makers can expect to profit personally if they deploy self-control and thus might be more likely to adjust their behaviour (even though people still struggle to resists the temptation of an immediate reward). Researchers have only recently started to study the intergenerational nature of sustainable decision-making by implementing consequences for a future generation into their experimental designs^14,15^: Corresponding to the theoretical idea that intergenerationally sustainable decision-making likely requires self-control capacity (and is thus prone to failure), it has been shown that social groups normally struggle to restrict their own consumption for the sake of a future generation. Note that concern for the future generation is not the only reason why people behave sustainably. For example, preserving the natural environment might be seen as value in and of itself by some people^16^. Still, the desire to benefit future generations is one of the key factors driving sustainable behaviour^17^.

Quite crucially, however, the idea that self-control is causally involved in intergenerationally sustainable decision-making, plausible as it might be, is not yet supported by strong data. In a first study, we could show that baseline activity in the right lateral PFC, a brain area known to be involved in self-control processes, is a stable predictor of sustainable behaviour^18^. This finding, however, is of correlational nature. To generate causal evidence in favour of the hypothesis that intergenerational sustainability relies on self-control capacity, one must experimentally modulate this capacity. One possible way of doing so would be the use of ego depletion tasks. However, the ongoing debate about the effectiveness of depletion procedures^19^ led us to choose a well-established brain stimulation approach. Here, we manipulated self-regulation capacity by applying inhibitory transcranial magnetic stimulation (TMS), a commonly employed brain modulation technique, on the right dorsolateral prefrontal cortex (dlPFC). There is ample evidence that inhibitory TMS on the right dlPFC reduces self-regulation capacity in social settings^20–25^. We employed a between-subject design with one group receiving the stimulation on the right dlPFC (henceforth: dlPFC group) and two control groups (see Materials and Methods for details).

**Figure 1:**
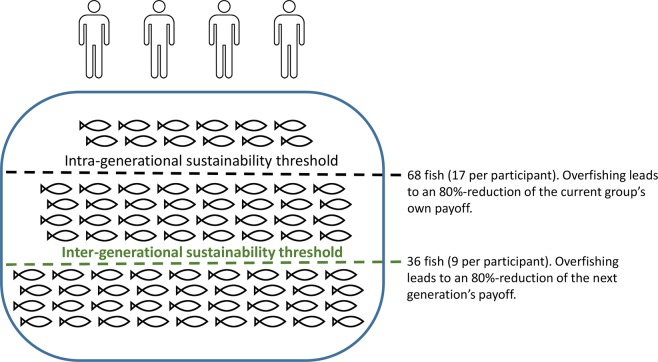
Schematic representation of the fishing game. The following rules apply: If the four participants in one group (i.e., generation) adhere to both the intra- and inter-generational sustainability threshold, extracting 36 fish or less (i.e., no more than an average of 9 fish per participant), both their own and the next generation receive the full payoff. If the participants adhere to the intra- but not the inter-generational sustainability threshold, extracting between 37 and 68 fish (no more than an average of 17 fish per participant), the payoff of the next generation is reduced by 80%. If the participants disregard both thresholds, extracting more than 68 fish (more than an average of 17 fish per participant), both their own payoff and that of the next generation is reduced by 80%.

To assess intergenerationally sustainable decision-making in a well-controlled laboratory setting, we used a newly developed intergenerational social dilemma game with an environmental framing, i.e., a fishing game. In this game, each participant can extract a maximum of 20 fish from a common pond (see Fig. 1 for a graphical representation). They played in groups of four, each group forming one generation in the lab. Participants receive a monetary reward for each extracted fish and their decisions directly affect the next people to come to the lab. Because this future group is completely unknown to the participants and any negative effects will happen with considerable delay, we can use behaviour towards the next group as proxy for behaviour towards the next generation. As in everyday life, if they want to behave sustainably towards a future generation, participants have to restrict themselves: If the collective extraction exceeds an *inter-generational sustainability threshold* of 36 fish, the next generation in the laboratory will not receive the full payoff from their extraction in the fishing game; their payoff will be reduced by 80%. This inter-generational sustainability threshold of 36 fish on a group level results in an individual inter-generational sustainability threshold of nine, i.e., on average, every participant can extract nine fish whilst still behaving sustainably towards the next generation. We hypothesised that self-control is involved in sustainable decision making and thus, we assumed that the extraction was larger when inhibiting the right dlPFC (i.e., participants will extract more fish, thereby behaving less sustainably, when self-control is weak). In everyday life, unsustainable decisions are not always equally appealing, thus requiring varying self-control effort. For example, it takes little effort to recycle glass bottles when the recycle bin is close to one’s home, but substantially more effort when recycling involves carrying the bottles for half a mile. To mirror this fact, we varied the exchange rate between one fish and the money participants received, thereby manipulating participants’ temptation to not behave sustainably. Since more self-control effort is required when the temptation is high, we assumed that diminishing self-control by means of inhibitory TMS on the right dlPFC would have the strongest effect on sustainable decision-making when the exchange rate was high.

We also wanted to ensure that participants could still behave rationally. Thus, our design includes another sustainability threshold: the *intra-generational sustainability threshold* of 68 fish (i.e., on average 17 fish per participant). If the collective extraction exceeded the intra-generational sustainability threshold, the participants lost 80% of the payoff from their extraction in the fishing game, making threshold-adherence personally relevant to the participants. Because of this significant potential personal loss, and because the intra-generation sustainability threshold was rather high, it would not be rational for participants to exceed this threshold. We thus expected that the three treatment groups should equally adhere to the intra-generational sustainability threshold.

## Results

**Figure 2:**
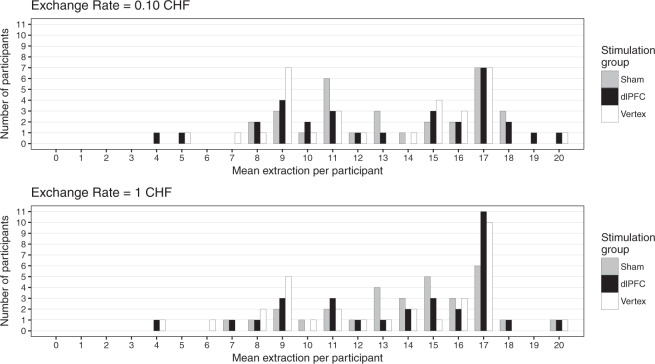
Mean extraction per participant and exchange rate, by stimulation group. The bars show the number of participants (y-axis) who (on average) extracted a specific amount of fish (x-axis). The colours represent the different stimulation groups; the two panels are separated for the two exchange rates. To not harm the next generation, a group had to restricted their catch to 36 fish (on average 9 per person); to not harm their own payoff, they had to restrict themselves to 68 fish (on average 17 per person).

Regardless of stimulation, participants’ mean extraction was well above the inter-generational threshold (see Table 1 for the descriptive results). While there was considerable variance within the extractions, participants’ extractions clustered at 17 fish (the intra-generational sustainability threshold) and around 9 fish (the inter-generational sustainability threshold), see Fig. 2 for a graphical depiction of the individual extractions. To test whether participants’ extraction was influenced by the exchange rate (1 CHF vs. 0.10 CHF) or inhibition of the right dlPFC (compared to vertex stimulation and sham stimulation), we performed a linear mixed-effects analysis with participants’ extraction per trial as dependent variable. As fixed effect, we entered exchange rate and stimulation group into the model (with 0.10 CHF resp. dlPFC group as baseline for all subsequent models), and added random intercepts for the subjects. Indeed, participants extracted more fish when one fish was worth 1 CHF (β = 0.07, SE = 0.023, p = 0.004, see Supplementary Material A), indicating less sustainable behaviour when the reward was large, as one would expect. However, there was no effect of TMS on extraction, as neither sham nor vertex-group showed statistically significant differences compared to the dlPFC group (β = −0.02, SE = 0.10, p = 0.87, and β = −0.08, SE = 0.10, p = 0.40, resp., see Supplementary Material A). In a next step, we added the interaction between exchange rate and stimulation group to the model. If a TMS-effect would only appear for one exchange rate or would take different directions or magnitudes depending on the exchange rate, this interaction would be statistically significant. However, this was not the case, neither for the sham-group nor the vertex-group (β = 0.01, SE = 0.041, p = 0.76, and β = −0.01, SE = 0.041, p = 0.77, resp., see Table 1 for the descriptive results, Fig. 2 for a graphical representation, and Supplementary Material A for details). As a comprehension check, participants had to indicate how many fish they could extract without reducing the payoff of the next generation after the experiment. To corroborate our finding, we calculated the same model with only those participants who answered correctly to this comprehension check (n = 74). Restricting our sample to this size did not alter the observed pattern, see Supplementary Material B.

**Table 1:** Mean (SD) of the extraction per stimulation group and exchange rate.

Stimulation Group	0.10 CHF	1 CHF
Right dlPFC	13.42 (4.15)	13.98 (3.67)
Sham	13.20 (4.49)	13.90 (4.31)
Vertex	12.73 (4.59)	13.15 (4.39)

### Control variables

To corroborate these findings, we wanted to control whether there were a priori differences between the stimulation groups in any relevant measure that could account for the absence of a TMS-effect. The online questionnaires employed covered pro-environmental attitudes, personal values, pro-social preferences, and trait self-control (see Methods for details), and 83 participants filled in the online questionnaires. We therefore added all questionnaires and participants’ age and gender as covariates to a model with stimulation group and exchange rate (and the interaction thereof in an additional model) as fixed effects, and random intercepts for the subjects. Because there is some evidence that cortical thickness can influence the effect of TMS^26^, we also calculated participants’ cortical thickness at the stimulation point and added it to the model (see Supplementary Material C). Including these covariates did not change the pattern of results reported above (see Supplementary Material D).

### Adherence to the inter-generation sustainability threshold

It could be argued that rather than using the raw extraction score as dependent variable, it is more meaningful to dichotomise participants’ decisions into sustainable and unsustainable ones. For example, an extraction of both 10 or 16 fish exceeds the individual inter-generation sustainable threshold, and an extraction of both 9 and 5 fish stays within its bounds. Thus, the first two decisions could be seen as not sustainable, the latter two as sustainable. However, when using the raw extraction as (linear) dependent variable, this important categorisation is lost: the extraction of 10 fish would be regarded as more closely related to an extraction of 9 fish than 16 fish, even though both 10 and 16 are unsustainable extractions. To circumvent this, we calculated how often each participant adhered to the inter-generation sustainability threshold (dichotomising their decisions), and repeated the analyses described above with this alternative and more precise measure of sustainability. The analyses with this new dependent variable did not reveal any significant effects of treatment group, neither as main effect (β = 0.04, SE = 0.11, p = 0.72 and β = 0.16, SE = 0.11, p = 0.15 for sham and vertex, respectively, see Supplementary Material E for all these and the subsequent analyses), nor in interaction with exchange rate (β = 0.03, SE = 0.09, p = 0.73 and β = 0.05, SE = 0.09, p = 0.54 for sham and vertex, respectively). Restricting the sample size to those 74 participants who answered correctly to the comprehension check did also not change the observed pattern, with no significant effect of exchange rate (β = −0.07, SE = 0.051, p = 0.196), stimulation group (β = −0.019, SE = 0.124, p = 0.873 and β = 0.142, SE = 0.124, p = 0.254 for sham and vertex), or the interaction thereof (β = 0.016, SE = 0.095, p = 0.864 and β = −0.100, SE = 0.095, p = 0.298 for sham and vertex, respectively). When including the previously discussed control variables, the effects were still not significant, see Supplementary Material E.

### Intra-generation sustainability threshold

Our design included not only the inter-generation sustainability threshold, but also the intra-generation sustainability threshold. This enabled us to ensure that participants could still behave rationally after TMS, i.e., that they were still able to observe a rule that was meaningful for their own payoff, even though they might behave unsustainable towards a future generation. Overall, exceeding the intra-generation threshold was rare and only occurred in 8% of decisions in the dlPFC group and sham group, and 7% of decisions in the vertex group. This speaks for the feasibility of our design: Indeed, participants seemed to be well aware whether their behaviour would hurt their own financial interest or “just” that of strangers in the future. For statistical analyses, see Supplementary Material F.

### Fairness rating

At the end of our experiment, participants had to answer the question which extraction they regarded as fair (for 0.10 and 1 CHF, respectively). To check whether TMS had an effect on the extraction participants deemed as fair, we calculated a mixed ANOVA with perceived fairness as dependent variable, exchange rate as within subject factor and stimulation group as between subject factor. There was no statistically significant effect of stimulation group, F(2,90) = 2.29, p = 0.11, exchange rate, F(1,90) = 2.87, p = 0.09, or the interaction thereof, F(2,90) = 0.12, p = 0.89.

## Discussion

We hypothesised that applying inhibitory TMS to the right dlPFC would lead to less sustainable decision-making towards a future generation. Our data do not comply with this hypothesis. This absence of a TMS-effect is robust when controlling for a broad range of relevant covariates. Of course, the absence of evidence for an effect is not the same as evidence for no effect. However, the high p-values, the absence of descriptive differences between the stimulation groups, and our large sample all lessen the probability of a Type II Error.

Our results led us to re-evaluate the appropriateness of our methodology. First, there might be important differences between sustainable behaviour in the real world and our model of sustainable behaviour in the lab. For example, in the real-world, cooperation of many more people is required to make a meaningful difference (compared to the four participants per group in our study). It might therefore be that our participants had higher perceived control over the situation than people have in real life. However, even if participants did have high perceived control, this did not result in especially sustainable behaviour, as our participants tended to overfish. Similarly, it could be that our design did not provide an adequate model of the next generation. It could be that in real life, people are more attached to the next generation, maybe because their own children and grandchildren are among this next generation. While this is possible, the observation that people feel rather detached from future generations at large^27^ makes this rather unlikely. With regard to the brain stimulations, it could be that the lack of effects is due to the fact that we stimulated the wrong part of the right dlPFC. However, the stimulation site was chosen based on a large number of previous studies, showing that self-control in a social setting can be altered by TMS to the right dlPFC e.g.^21,24^, and we used MRI-guided neuronavigation to make sure to target the correct area. Another possibility would be that our stimulation did not have the desired physiological effect. Indeed, there seem to be genetic factors (specifically, a variant of the brain-derived neurotrophic factor) that influence whether a person is susceptible to brain stimulation^28^. However, it seems unlikely that our sample consisted exclusively or almost exclusively of carriers of this particular allele, especially given our large sample size. Similarly, there is some evidence that cTBS can have both excitatory and inhibitory effects, especially in 50-Hz-cTBS, even though this has so far only been shown in motor areas^29^. Yet, it has also been shown that in comparison to the 50-Hz-protocol, the 30-Hz-protocol used in our study has consistently large inhibitory effects^30^. Another explanation for no effect might be an a priori difference in cortical thickness, as it has been argued that cortical thickness at the stimulation site might influence the effectiveness of TMS in certain cases^26^. However, when controlling for differences in cortical thickness at the right dlPFC, we still found no effect. It therefore seems unlikely that our results are a statistical or methodological artefact. Rather, we must assume that in the specific situation modelled in our experiment (where participants could restrict their own payoff for the sake of strangers in the future) inhibition of the right dlPFC does not alter participants’ behaviour. Below, we will outline the most plausible reasons for this finding. First, while self-control is most prominently associated with the right dlPFC, it is far from being the only brain region involved in self-control processes. It could be that other self-control related brain regions like the vlPFC were involved in the decisions of our participants and enabled them to exert enough self-control to suppress their selfish impulses for the sake of a future generation. Similarly, one could wonder whether we were correct in stimulating the right, and not the left dlPFC. This is a valid objection, especially given prior evidence that inhibiting the left dlPFC does lead to less self-control, for example in the context of intertemporal choice^13^. However, given the vast literature showing that the inhibition of the right dlPFC is sufficient to diminish self-control in social situations^20–25,31^, we do not believe that inhibiting the left dlPFC instead of the right dlPFC would have been a more feasible approach to diminish self-control in our paradigm.

Alternatively, it could be that we did not observe an effect of inhibiting the right dlPFC because our scenario put a strong focus on the next generation. While in people’s daily life, the fact that there will be future generations after us often receives little attention, the next generation was mentioned on every decision screen in our experiment. This salient and prominent role of the next generation might have had substantial influence and altering participants’ social cognition might have shown a stronger behavioural effect. For example, one could speculate whether applying inhibitory TMS to the TPJ or the mPFC, areas associated with social cognition^32,33^, would have impaired participants’ ability to take the perspective of other participants in the future, thereby leading to less sustainable decision-making towards a future generation. Indeed, recent evidence shows that a failure to act sustainably can be seen as temporal “intergroup bias” and encouraging participants to come up with similarities between the current and future generation makes them more sustainable^27^. Because of the known relation between the TPJ and intergroup bias^34^, one could assume that in situations where the next group is saliently perceived as an in- or outgroup, the TPJ’s involvement is especially important – even more so as disrupting the TPJ leads to less prosocial behaviour and to a stronger focus on immediate rewards^35^.

Summing up, we could not find evidence that disrupting the right dlPFC (which has previously been shown to reduce self-control) had any effect on sustainable behaviour towards a future generation. This is at odds with recent theorising in environmental psychology and recent correlational findings. In the future, it might be worth exploring whether there is a subset of situations in which sustainable behaviour does or does not require self-control effort, whether there are situations in which social cognition is more relevant, and how these respective situations are characterised.

## Methods

### Participants

Participants consisted of 100 right-handed students of the University of Bern with normal or corrected-to-normal vision. The data of 3 participants were not properly recorded due to a computer problem. During the experiment, two subjects stated that they did not want to receive the TMS. Two subject appeared to be intoxicated, their data was not further analysed. Hence, our final sample consists of 93 participants (49 females, mean age 22.1, SD = 2.39). Participants gave written informed consent before participation in the study, which had been approved by the cantonal ethics committee Bern (KEK Bern; No. 2017-01166) and was conducted in accordance to both the declaration of Helsinki and all relevant guidelines and regulations. Students of psychology, economics, and social sciences were not admitted to the experiment, as they might have encountered similar experimental designs during their studies and might hence behave differently from naïve subjects. Those with a history of mental or neurological disorder were not admitted to the experiment. See Supplementary Material G for a detailed description of the exclusion criteria. Participants never took part in a TMS-experiment or an economic game in our lab. Only one subject had prior experience with TMS.

### Procedure

Participants were pseudorandomly distributed to one of three groups (controlling for gender): The first group (dlPFC group; 31 participants; 17 female) received the inhibitory TMS protocol on the right dlPFC (producing after-effects of at least 30 minutes^36–38^), the second group (sham group; 31 participants; 15 female) received the same TMS protocol with a sham coil on the right dlPFC, and the third group (vertex group; 31 participants; 17 female) received the same TMS protocol on the vertex as an active control site (see below for details). Upon arrival, participants’ individual resting motor threshold was determined and the stimulation site was marked on their head, using a stereotaxic infrared neuronavigation system (see below for details). Next, all participants received the instructions for the fishing game and had to answer four questions about the game to ensure they correctly understood the rules. Participants then received the inhibitory TMS either at the right dlPFC, the vertex, or sham TMS at the dlPFC, depending on their group (see below for details on the stimulation procedure). After its application, participants played the fishing game (see below). Directly following the fishing game, they were asked to rate which behaviour in the game they would deem fair (see below). Participants were then asked to state how many fish each participant could have extracted on average while still acting sustainably (see below). To minimise undesired social effects between participants, they were physically separated during neuronavigation, determination of motor threshold, and application of TMS, so that no interaction took place before the fishing game. One week after the TMS session, participants received a link to three questionnaires (see below) via email and were asked to fill them out online. This delayed assessment was implemented to ensure that TMS has no effect on participants’ responses in the questionnaires.

### Acquisition of brain anatomy

Anatomical data were acquired on a Siemens Prisma 3.0 Tesla whole-body scanner (Siemens Erlangen) using a 64-channel head coil. T1-weighted 3D modified driven equilibrium Fourier transformation (MDEFT) images were acquired from each subject (176 slices, Field of View: 256 × 256 × 176 mm, slice thickness: 1 mm, no gap, repetition time: 7.93 ms, echo time: 2.49 ms, flip angle: 16°).

### TMS protocol

We applied continuous theta burst stimulation (cTBS) before participants took part in an intergenerational sustainability dilemma (fishing game, see next section). We used a cTBS protocol^36^, consisting of a single cTBS train with 801 pulses organised in 267 bursts, each burst containing three pulses at 30 Hz, with an interburst interval of 100 ms and stimulation intensity at 80% of participants’ resting motor threshold. The resting motor threshold was defined as the intensity at which a stimulation of the motor cortex region corresponding to the left index finger elicited a visually observable movement 5 out of 10 times. The dlPFC group received cTBS on the right dlPFC, the vertex group received cTBS on the vertex, the sham group received cTBS with a sham coil.

To localise the stimulation sites, we acquired individual anatomical MR images (T1-weighted) for each participant. These brain images were used to guide the stimulation using the stereotaxic infrared system Localite NeuroNavigator (Localite, St. Augustin, Germany). The stimulation site [Talairach coordinates: *x* = 39, *y* = 37, *z* = 22] follows prior studies on self-control capacity and the right dlPFC^21,24,39^. The vertex was defined as the point on the scalp that lay halfway between nasion and inion resp. the left and right tragus. Both dlPFC and vertex were marked in the brain images so that the experimenter could then navigate to the appropriate position. The coil was held on the participant’s head with the handle pointing caudally. CTBS was applied using a MagPro X100 stimulator (MagVenture, Farum, Denmark) with a figure-of-eight coil (MC-B70, inner radius 27 mm, outer radius 97 mm) resp. the corresponding sham coil (MC-P-B70).

### Assessment of intergenerationally sustainable decision-making

To assess the sustainability of participants’ decision making, they engaged in a computerised fishing game (implemented using z-tree^40^) with real, monetary consequences for both their own generation as well as the subsequent group (generation) in the laboratory. Participants were asked to take the role of a fisher who can extract between 0 and 20 fish from a common pool shared with three other participants. Depending on the round, one fish was worth either 1 CHF or 0.10 CHF (varying the temptation participants face); participants were informed about the current value before every decision. Participants were informed that at the end of the experiment, two trials would be selected by a random mechanism (one trial with a fish worth 1 CHF and one trial with a fish worth 0.10 CHF) and the amount of money corresponding to their extraction would be paid to them in cash (in addition to a flat fee of 55 CHF). If the four participants collectively extracted more than 36 fish (the inter-generational sustainability threshold), the payoff in that trial was reduced by 80% for the next group of four (i.e., generation) to come to the lab. Similarly, if the participants collectively extracted more than 68 fish (the intra-generational sustainability threshold) in any given trial, their payoff from that trial was reduced by 80%. This was implemented to ensure that TMS on the right dlPFC leaves participants still able to understand the consequences of their actions, as we did not expect that the higher intra-generational sustainability threshold that has potentially negative consequences for one’s own payoff would be exceeded more often after TMS on the right dlPFC compared to sham-TMS or TMS on the vertex.

Participants were asked to complete eight trials in a pseudo-randomised order with four trials per exchange rate (i.e., one extracted fish is either worth 1 CHF or 0.10 CHF). Each trial consisted of one decision screen: Participants saw which exchange rate applied, and were reminded about the inter- and intra-generational sustainability thresholds. They saw the question “How many fish do you want to extract” at the bottom of the screen, and a visual scale with the numbers from 0 to 20. They made their decision by clicking on the appropriate number, which was highlighted shortly afterwards. Participants of the same generation made their decisions at the same time and in the same room, but anonymously and without visual contact. Additionally, they were instructed not to speak to each other.

Before the experiment, participants were informed that they would receive their payment with no other person present and from a person who could not infer their decisions from their payoff. This was done to eliminate reputation effects towards the experimenter or other participants. Additionally, feedback on the collective extraction of their other group members, their payoff, and the consequences of their behaviour for the next generation was only provided once at the very end of the experiment, so that it could not influence participants’ decision.

### Fairness ratings

To rule out the possibility that a TMS effect was due to changes in perceived fairness rather than self-control capacity, participants were asked to rate which extraction they see as fair while still under the influence of TMS. For this, they were reminded of the two types of decision screens (with one fish worth 1 CHF resp. 0.10 CHF) and were asked to judge which amount of extracted fish they see as fair (on a scale from 0 to 20, as in the actual fishing game). This was done after the fishing game to prevent this question from priming a certain behaviour during the actual decision-making.

### Inter-generational threshold comprehension check

To rule out the possibility that a possible TMS effect was due to changes in task understanding rather than self-control capacity, participants were asked to state the average amount of fish each player could extract so that the group would not exceed the inter-generational sustainability threshold (on a scale from 0 to 20) while they were still under the influence of TMS. Hence, this question allows us to ensure that participants did not accidently behave unsustainably while believing they were behaving sustainably (or vice versa).

### Questionnaires

We wanted to ensure that potential TMS effects were not due to stable differences between the treatment groups with regard to variables that could influence participants’ extraction in the fishing game (e.g. environmental attitudes or pro-social preferences). Hence, one week after the experiment, participants were asked to fill out four online questionnaires. To measure environmental attitudes and personal values (including biospheric values, i.e., concern for nature, and egoistic values), we used the well-established New Environmental Paradigm^16^ and the Schwartz Value Scale^41^. To assess participants’ pro-social preferences, we used the Honesty-Humility Scale from the Hexaco^42^. Finally, to control for differences in trait self-control, we used the short version of the self-control scale by Tagney, Baumeister, & Boone^43^.

### Statistical analyses

To analyse our data, we ran mixed-effect models. Mixed-effect models allow to account for both fixed effects (e.g., differences between treatment groups) and random effects (e.g., trial-by-trial variation for each subject). Here, we first tested for main effects of TMS group and exchange rate on participants’ extraction, and then added the interaction of the two to see whether a TMS-effect was specific to only one exchange rate. Finally, we corroborated our results by including relevant covariates and additional control measures, please refer to the results section for details. Any effect was regarded as statistically significant if p < 0.05 (two-tailed). For all mixed-effect models, visual inspection of residual plots did not reveal any obvious deviations from homoscedasticity or normality. Analyses were conducted using the statistic software R^44^, mixed-effects models were calculated using the R-package lme4^45^.

## Supplementary information

Supplementary Information
