# Supplementary material for: Inhibition of the right dlPFC by theta burst stimulation does not alter sustainable decision-making
Source: Sci Rep. 2019 Sep 25;9:13852. doi: 10.1038/s41598-019-50322-w (PMC6761266; doi:10.1038/s41598-019-50322-w)
Supplement: Supplementary file 1 — Supplementary Information [file 41598_2019_50322_MOESM1_ESM.docx]

**Supplementary Material**

**Inhibition of the right dlPFC by theta burst stimulation does not alter sustainable decision-making**

Benedikt P. Langenbach^1+^, Thomas Baumgartner^1+^, Dario Cazzoli^2^, René M. Müri^2^, Daria Knoch^1*^

1: University of Bern, Institute of Psychology, Department of Social Psychology and Social Neuroscience, Fabrikstrasse 8, 3012 Bern, Switzerland

2: University Hospital Bern, Department of Neurology, University Neurorehabilitation, Freiburgstrasse 10, 3010 Bern, Switzerland

^+^ The first two authors contributed equally to this work

^*^Correspondence should be addressed to Daria Knoch:

Department of Social Psychology and Social Neuroscience
Institute of Psychology
University of Bern
Fabrikstrasse 8
3012 Bern
Switzerland
tel: 0041 31 631 4690
mail: daria.knoch@psy.unibe.ch

**Supplementary Material A**

| *Table A1: Mixed-effects model with stimulation group (baseline: right dlPFC), exchange rate (baseline: 0.10 CHF) as predictors, extraction as dependent variable, and random intercepts for subjects.* | | | | |
| --- | --- | --- | --- | --- |
| Predictor | β | Standard Error | Df | p |
| Sham stimulation | -0.016 | 0.099 | 90 | .871 |
| Vertex stimulation | -0.084 | 0.099 | 90 | .398 |
| Exchange rate 1 CHF | 0.065 | 0.023 | 650 | .004 |

*n = 93
REML criterion at convergence: 3797.1*

| *Table A2: Mixed-effects model with stimulation group (baseline: right dlPFC), exchange rate (baseline: 0.10 CHF), and their interaction as predictors, extraction as dependent variable, and random intercepts for subjects. Note that in a regression model with an interaction term, the individual estimates of the predictors included in the interaction term are only valid for the case that the interaction is zero. Thus, the estimates of “Sham stimulation” and "Vertex stimulation" are only valid for the exchange rate of 0.10 CHF. Similarly, the estimate of "Exchange rate 1 CHF" is only valid for the dlPFC stimulation group. The important aspects of this model, however, are the results of the interactions.* | | | | |
| --- | --- | --- | --- | --- |
| Predictor | β | Standard Error | Df | p |
| Sham stimulation | -0.024 | 0.102 | 103 | .815 |
| Vertex stimulation | -0.076 | 0.102 | 103 | .456 |
| Exchange rate 1 CHF | 0.065 | 0.039 | 648 | .098 |
| Interaction Sham stimulation and exchange rate 1 CHF | 0.013 | 0.041 | 648 | .760 |
| Interaction Vertex stimulation and exchange rate 1 CHF | -0.012 | 0.041 | 648 | .773 |

*n = 93
REML criterion at convergence: 3796.4*

**Supplementary Material B**At the end of the experiment, participants were asked how many fish every participant could have extracted on average before the payoff of the next group in the lab was reduced. Out of our sample of 93 participants, 19 answered incorrectly to this question (even though most only deviated slightly, with 11 people only being one fish off). To make sure that those people did not influence our results, we restricted our sample to the 74 participants who had answered correctly. However, this did not change the pattern of results: We did find a significant main effect of exchange rate (β = .064, SE = .026, p = .016, see table B1), but no effect of stimulation group (β = .039, SE = .110, p = .727 and β = -.088, SE = .110, p = .428 for sham and vertex, respectively, see table B1), and no effect of the interaction thereof (β = .018, SE = .049, p = .717 and β = -.039, SE = -.049, p = .436 for sham and vertex, respectively, see table B2).

| *Table B1: Mixed-effects model with stimulation group (baseline: right dlPFC), exchange rate (baseline: 0.10 CHF) as predictors, extraction as dependent variable, and random intercepts for subjects (restricted sample).* | | | | |
| --- | --- | --- | --- | --- |
| Predictor | β | Standard Error | Df | p |
| Sham stimulation | 0.039 | 0.110 | 71 | .727 |
| Vertex stimulation | -0.088 | 0.110 | 71 | .428 |
| Exchange rate 1 CHF | 0.064 | 0.026 | 517 | .016 |

*n = 74
REML criterion at convergence: 3039.4*

| *Table B2: Mixed-effects model with stimulation group (baseline: right dlPFC), exchange rate (baseline: 0.10 CHF), and their interaction as predictors, extraction as dependent variable, and random intercepts for subjects (restricted sample). Note that in a regression model with an interaction term, the individual estimates of the predictors included in the interaction term are only valid for the case that the interaction is zero. Thus, the estimates of “Sham stimulation” and "Vertex stimulation" are only valid for the exchange rate of 0.10 CHF. Similarly, the estimate of "Exchange rate 1 CHF" is only valid for the dlPFC stimulation group. The important aspects of this model, however, are the results of the interactions.* | | | | |
| --- | --- | --- | --- | --- |
| Predictor | β | Standard Error | Df | p |
| Sham stimulation | 0.027 | 0.114 | 83 | .812 |
| Vertex stimulation | -0.064 | 0.114 | 83 | .580 |
| Exchange rate 1 CHF | 0.074 | 0.047 | 515 | .121 |
| Interaction Sham stimulation and exchange rate 1 CHF | 0.018 | 0.049 | 515 | .717 |
| Interaction Vertex stimulation and exchange rate 1 CHF | -0.039 | 0.049 | 515 | .436 |

*n = 74
REML criterion at convergence: 3037*

**Supplementary Material C**

As explained in the main manuscript, there is some evidence suggesting that cortical thickness at the stimulation area might influence the effect of TMS. In order to check whether this was the case in our experiment, we analysed cortical thickness with the computational anatomy toolbox (CAT 12, version r1318) implemented in the statistical parametric mapping software (SPM 12, version 7219). Preprocessing of the data involved spatial normalization to a DARTEL MNI template, segmentation into gray matter (GM), white matter (WM), and cerebrospinal fluid (CSF) [1, 2]. CAT 12 estimates cortical thickness and central surface (created at the 50% distance boundary between GM/WM and GM/CSF) based on the projection-based thickness method [3]. This method uses tissue segmentation to estimate the WM distance and then projects the local maxima (which is equal to the cortical thickness) onto other GM voxels using a neighbouring relationship described by the WM distance. This projection-based thickness method allows the handling of partial volume information, sulcal blurring, and sulcal asymmetries without explicit sulcus reconstruction. The reconstruction of the central surface includes topology correction, which accounts for topological defects using spherical harmonics [4]. Furthermore, spherical mapping is applied to reparametrize the surface mesh into a common coordinate system [5], while spherical registration adapts the volume-based diffeomorphic DARTEL algorithm to the surface [6]. In order to extract cortical thickness values in the area of the right dlPFC, we created a spherical ROI (10 mm diameter) around the stimulated peak voxel using the WFU Pick Atlas. Then we mapped this spherical ROI from volume space to surface space (32k mesh). This surface ROI (see Figure C1) was then used to extract average cortical thickness values in the area of the right dlPFC. The extracted ROI data were then transferred to statistical tools to analyse whether cortical thickness had an effect when included as a covariate in the relevant mixed effect models (for details please see result section of the main manuscript).


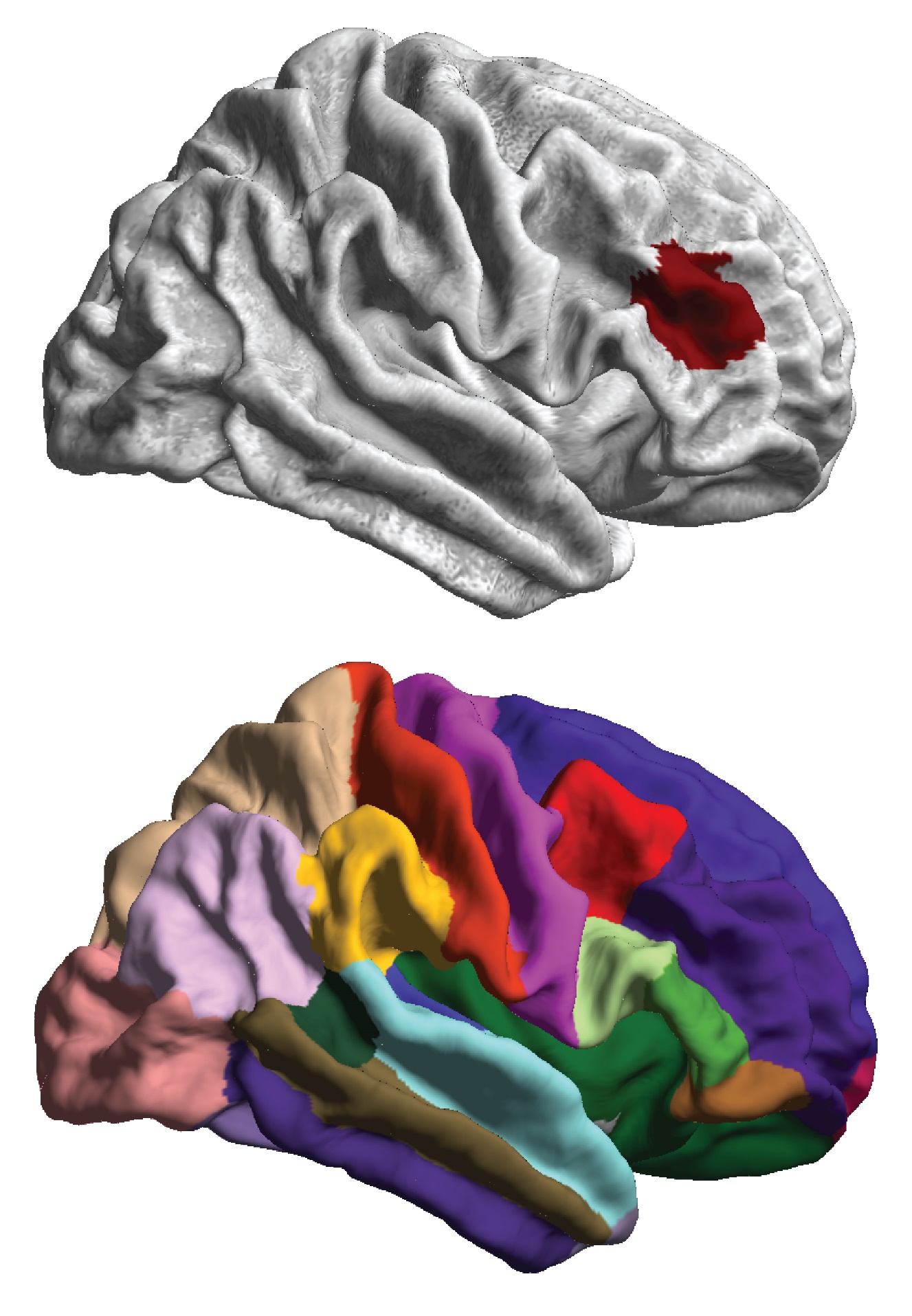


Figure C1: Surface ROI around the stimulated coordinate [x = 39, y = 37, z = 22].

1. J. Ashburner (2005): Unified segmentation. Neuroimage 26(3):839-51.
2. J. Ashburner (2007): A fast diffeomorphic image registration algorithm. Neuroimage 38(1):95-113.
3. Dahnke R, Yotter RA, Gaser C. Cortical thickness and central surface estimation. Neuroimage 2013;65:336-48.
4. Yotter RA, Dahnke R, Thompson PM, et al. Topological correction of brain surface meshes using spherical harmonics. Hum Brain Mapp 2011;32:1109-24.
5. Yotter RA, Thompson PM, Gaser C. Algorithms to improve the reparameterization of spherical mappings of brain surface meshes. J Neuroimaging 2011;21:e134-47.
6. Ashburner J. A fast diffeomorphic image registration algorithm. Neuroimage 2007;38:95-113.

**Supplementary Material D**As discussed in the main manuscript, we included a number of relevant covariates into our analysis. However, their inclusion did not change the pattern of results, neither for the model with only the main effects (see table D1) nor for the model with the interaction between stimulation group and exchange rate (see table D2). 83 participants filled in the online questionnaires, the descriptive statistics are reported in table D3.

| *Table D1: Mixed-effects model with stimulation group (baseline: right dlPFC) and exchange rate (baseline: 0.10 CHF) and relevant covariates as predictors, extraction as dependent variable, and random intercepts for subjects.* | | | | |
| --- | --- | --- | --- | --- |
| Predictor | β | Standard Error | Df | p |
| Sham stimulation | .003 | .115 | 70 | .979 |
| Vertex stimulation | -.062 | .109 | 70 | .569 |
| Exchange rate 1 CHF | .07 | .024 | 580 | .002 |
| Gender: Female | -.183 | .095 | 70 | .059 |
| Age | .057 | .105 | 70 | .592 |
| Egoism | .097 | .107 | 70 | .368 |
| Altruism | .144 | .109 | 70 | .191 |
| Biospherism | -.235 | .141 | 70 | .101 |
| Hedonism | .147 | .106 | 70 | .169 |
| NEP | .140 | .128 | 70 | .276 |
| Self-Control | .130 | .096 | 70 | .180 |
| Honesty-Humility | -.075 | .108 | 70 | .491 |
| Cortical thickness of right dlPFC | .028 | .097 | 70 | .778 |

*n = 83
REML criterion at convergence: 3385.1*

| *Table D2: Mixed-effects model with stimulation group (baseline: right dlPFC), exchange rate (baseline: 0.10 CHF), their interaction, and relevant covariates as predictors, extraction as dependent variable, and random intercepts for subjects. Note that in a regression model with an interaction term, the individual estimates of the predictors included in the interaction term are only valid for the case that the interaction is zero. Thus, the estimates of “Sham stimulation” and "Vertex stimulation" are only valid for the exchange rate of 0.10 CHF. Similarly, the estimate of "Exchange rate 1 CHF" is only valid for the dlPFC stimulation group. The important aspects of this model, however, are the results of the interactions.* | | | | |
| --- | --- | --- | --- | --- |
| Predictor | β | Standard Error | Df | p |
| Sham stimulation | -.013 | .118 | 79 | .916 |
| Vertex stimulation | -.070 | .112 | 79 | .536 |
| Exchange rate 1 CHF | .056 | .043 | 578 | .188 |
| Interaction sham stimulation and exchange rate 1 CHF | .025 | .045 | 578 | .580 |
| Interaction vertex stimulation and exchange rate 1 CHF | .012 | .045 | 578 | .787 |
| Gender: Female | -.183 | .095 | 70 | .059 |
| Age | .057 | .105 | 70 | .592 |
| Egoism | .097 | .107 | 70 | .368 |
| Altruism | .144 | .109 | 70 | .191 |
| Biospherism | -.235 | .141 | 70 | .101 |
| Hedonism | .147 | .106 | 70 | .170 |
| NEP | .140 | .128 | 70 | .276 |
| Self-Control | .130 | .096 | 70 | .180 |
| Honesty-Humility | -.075 | .108 | 70 | .492 |
| Cortical thickness of right dlPFC | .028 | .097 | 70 | .778 |

*n = 83*
*REML criterion at convergence: 3385.1*

| *Table D3: Descriptive results (mean & SD) of the online questionnaires, separate for those participants that received stimulation at the dlPFC at the vertex and sham stimulation at the dlPFC. For the Honesty-Humility-Scale and the NEP, participant’s answered on a 5-point Likert-Scale from “strongly agree” to “strongly disagree”; for the Self-Control-Scale, they answered on a 5-point Likert-Scale from “Not at all” to “Very Much”, for the subscales of the Schwartz’ Value Scale (Egoism, Altruism, Biospherism, Hedonism) they rated each value on an 8-point Likert-Scale ranging from “opposed to my values” to “extremely important for me”.* | | | |
| --- | --- | --- | --- |
|  | dlPFC group | Sham group | Vertex group |
| NEP | 3.78 (0.40) | 3.71 (0.39) | 3.78 (0.39) |
| Self-Control | 2.94 (0.65) | 3.04 (0.68) | 3.19 (0.57) |
| Honesty-Humility | 3.49 (0.56) | 3.51 (0.48) | 3.55 (0.44) |
| Hedonism | 6.33 (0.86) | 6.42 (1.02) | 6.49 (0.88) |
| Altruism | 6.61 (0.80) | 6.30 (0.98) | 6.59 (1.11) |
| Biospherism | 6.39 (0.95) | 6.06 (1. 07) | 6.45 (1.07) |
| Egoism | 4.32 (1.19) | 3.94 (0.87) | 4.07 (0.89) |

**Supplementary Material E**

As described in the main manuscript, we dichotomised participants’ decisions into sustainable and unsustainable decisions (i.e., decisions that did exceed the individual inter-generation sustainability threshold and those that did not), and used the sum of sustainable decisions as dependent variable. Again, we found no statistically significant effect of exchange rate (β = ‑.044, SE = .047, p = .352), stimulation group (β = .038, SE = .107, p = .723 and β = .157, SE = .107, p = .145 for sham and vertex, respectively, see table E1), or the interaction thereof (β = .030, SE = .086, p = .728 and β = .053, SE = .086, p = .543 for sham and vertex, respectively, see table E2). Restricting the sample size to those 74 participants who answered correctly to the comprehension check did also not change the observed pattern, with no significant effect for exchange rate (β = -.066, SE = .051, p = .196), stimulation group (β = -.019, SE = .124, p = .873 and β = .142, SE = .124, p = .254 for sham and vertex, respectively, see table E3), or the interaction thereof (β = .016, SE = .095, p = .864 and β = -.100, SE = .095, p = .298 for sham and vertex, respectively, see table E4). When including the control variables discussed in the main text, the effects were still not significant, see table E5.

| *Table E1: Mixed-effects model with stimulation group (baseline: right dlPFC) and exchange rate (baseline: 0.10 CHF) as predictors, threshold adherence as dependent variable, and random intercepts for subjects.* | | | | |
| --- | --- | --- | --- | --- |
| Predictor | β | Standard Error | Df | p |
| Sham stimulation | 0.038 | 0.107 | 90 | .723 |
| Vertex stimulation | 0.157 | 0.107 | 90 | .145 |
| Exchange rate 1 CHF | -0.044 | 0.047 | 92 | .352 |

*n = 93*
*REML criterion at convergence: 662.6*

| *Table E2: Mixed-effects model with stimulation group (baseline: right dlPFC), exchange rate (baseline: 0.10 CHF) and their interaction as predictors, threshold adherence as dependent variable, and random intercepts for subjects. Note that in a regression model with an interaction term, the individual estimates of the predictors included in the interaction term are only valid for the case that the interaction is zero. Thus, the estimates of “Sham stimulation” and "Vertex stimulation" are only valid for the exchange rate of 0.10 CHF. Similarly, the estimate of "Exchange rate 1 CHF" is only valid for the dlPFC stimulation group. The important aspects of this model, however, are the results of the interactions.* | | | | |
| --- | --- | --- | --- | --- |
| Predictor | β | Standard Error | Df | p |
| Sham stimulation | 0.019 | 0.120 | 134 | .874 |
| Vertex stimulation | 0.124 | 0.120 | 134 | .304 |
| Exchange rate 1 CHF | -0.081 | 0.082 | 90 | .326 |
| Interaction sham stimulation and exchange rate 1 CHF | 0.030 | 0.086 | 90 | .728 |
| Interaction vertex stimulation and exchange rate 1 CHF | 0.053 | 0.086 | 90 | .543 |

*n = 93
REML criterion at convergence: 662.8*

| *Table E3: Mixed-effects model with stimulation group (baseline: right dlPFC) and exchange rate (baseline: 0.10 CHF) as predictors, threshold adherence as dependent variable, and random intercepts for subjects (restricted sample).* | | | | |
| --- | --- | --- | --- | --- |
| Predictor | β | Standard Error | Df | p |
| Sham stimulation | -0.019 | 0.124 | 71 | .873 |
| Vertex stimulation | 0.142 | 0.124 | 71 | .254 |
| Exchange rate 1 CHF | -0.066 | 0.051 | 73 | .196 |

*n = 74
REML criterion at convergence: 530.1*

| *Table E4: Mixed-effects model with stimulation group (baseline: right dlPFC), exchange rate (baseline: 0.10 CHF) and their interaction as predictors, threshold adherence as dependent variable, and random intercepts for subjects (restricted sample). Note that in a regression model with an interaction term, the individual estimates of the predictors included in the interaction term are only valid for the case that the interaction is zero. Thus, the estimates of “Sham stimulation” and "Vertex stimulation" are only valid for the exchange rate of 0.10 CHF. Similarly, the estimate of "Exchange rate 1 CHF" is only valid for the dlPFC stimulation group. The important aspects of this model, however, are the results of the interactions.* | | | | |
| --- | --- | --- | --- | --- |
| Predictor | β | Standard Error | Df | p |
| Sham stimulation | -0.302 | 0.137 | 103 | .827 |
| Vertex stimulation | 0.080 | 0.137 | 103 | .564 |
| Exchange rate 1 CHF | -0.120 | 0.091 | 71 | .195 |
| Interaction sham stimulation and exchange rate 1 CHF | 0.016 | 0.095 | 71 | .864 |
| Interaction vertex stimulation and exchange rate 1 CHF | 0.100 | 0.095 | 71 | .298 |

*n = 74
REML criterion at convergence: 529*

| *Table E5: Mixed-effects model with stimulation group (baseline: right dlPFC), exchange rate (baseline: 0.10 CHF), their interaction, and relevant covariates as predictors, threshold adherence as dependent variable, and random intercepts for subjects. Note that in a regression model with an interaction term, the individual estimates of the predictors included in the interaction term are only valid for the case that the interaction is zero. Thus, the estimates of “Sham stimulation” and "Vertex stimulation" are only valid for the exchange rate of 0.10 CHF. Similarly, the estimate of "Exchange rate 1 CHF" is only valid for the dlPFC stimulation group. The important aspects of this model, however, are the results of the interactions.* | | | | |
| --- | --- | --- | --- | --- |
| Predictor | β | Standard Error | Df | p |
| Sham stimulation | -.019 | .142 | 100 | .893 |
| Vertex stimulation | .122 | .136 | 100 | .372 |
| Exchange rate 1 CHF | -.049 | .092 | 80 | .592 |
| Interaction sham stimulation and exchange rate 1 CHF | .003 | .095 | 80 | .978 |
| Interaction vertex stimulation and exchange rate 1 CHF | .020 | .095 | 80 | .832 |
| Gender: Female | .107 | .106 | 70 | .318 |
| Age | -.107 | .117 | 70 | .363 |
| Egoism | -.082 | .119 | 70 | .496 |
| Altruism | -.196 | .122 | 70 | .113 |
| Biospherism | .122 | .158 | 70 | .442 |
| Hedonism | -.112 | .118 | 70 | .346 |
| NEP | -.068 | .143 | 70 | .638 |
| Self-Control | -.111 | .108 | 70 | .306 |
| Honesty-Humility | .087 | .121 | 70 | .474 |
| Cortical thickness of right dlPFC | -.023 | .109 | 70 | .830 |

*n = 67
REML criterion at convergence: 592.6*

**Supplementary Material F**

To statistically test for differences between the stimulation groups with regards to the intra-generation sustainability threshold, we calculated how often every participant adhered to the intra-generation sustainability threshold (separated for the two exchange rates) and used this value as dependent variable. When calculating a mixed model with stimulation group and exchange rate as predictors, no significant main effect emerged, neither for exchange rate (β = .037, SE = .049, p = .449), nor for stimulation group (β = .008, SE = .107, p = .935, and β = .035, SE = .107, p = .745, for sham and vertex, resp.). When adding the interaction between exchange rate and stimulation group to the model, this, too, did not reach statistical significance (β = .014, SE = .090, p = .878, and β = .027, SE = .090, p = .759, for sham and vertex, resp.).

**Supplementary Material G**Participants were screened in a telephone interview, exclusion criteria were implemented to ensure that participants could undergo both TMS and MRI without unnecessary risks. The specific exclusion criteria were:

- Studies of psychology or economics
- Impaired vision (if not corrected to normal)
- Left-handedness
- Claustrophobia
- Tattoos on the head/neck
- Piercings that could not be removed
- Dental braces
- Other metal in the body (e.g. artificial joints, implanted electrodes, copper coil)
- Pregnancy or breast-feeding
- Medical implant (e.g., pacemaker, insulin pump, artificial heart valve)
- Dental prosthesis or hearing aid
- Migraine
- Present or past neurological disorder (e.g., epilepsy, stroke, tumour)
- History of one or more seizures
- Present or past mental disorder (e.g., depression, substance abuse, schizophrenia)
- Cardiovascular disease
- Regular intake of (legal or illegal drugs) [if participants listed a drug, the responsible physician judged whether this drug could lower the seizure threshold or have otherwise negative interactions with TMS]
- History of operation at heart or head
- Injury at the head with loss of consciousness
